# Spatial memory in Alzheimer’s disease 5XFAD mice is enhanced by XPO1 inhibitor KPT-330

**DOI:** 10.1007/s11357-026-02122-0

**Published:** 2026-02-06

**Authors:** Shi Quan Wong, Adia Ouellette, Laura Harrison, Avery McNamara, Rachel A. Tam, Alexander Alexandrov, Acacia Nawrocik-Madrid, Jesus J. Sanchez, Brett C. Ginsburg, Arturo A. Andrade, Louis R. Lapierre

**Affiliations:** 1 Department of Molecular Biology, Cell Biology and Biochemistry, Brown University, 185 Meeting Street, Providence, RI 02912 USA; 2 Centre de Médecine de Précision du Nouveau-Brunswick, 27 Rue Providence, Moncton, NB E1C 8X3 Canada; 3 Département de Chimie et Biochimie, Université de Moncton, 18 Avenue Antonine Maillet, Moncton, NB E1A 3E9 Canada; 4 Analytical Pharmacology and Drug Evaluation Core, Nathan Shock Center San Antonio, University of Texas in San Antonio, 7703 Floyd Curl Drive, San Antonio, TX 78229 USA; 5 Department of Neuroscience, Brown University, 185 Meeting Street, Providence, RI 02912 USA; 6 Carney Institute for Brain Science, Brown University, 164 Angell Street, Providence, RI 02912 USA

**Keywords:** Alzheimer’s disease, XPO1, KPT-330

## Abstract

The proteostatic decline in Alzheimer’s disease is well established, and improvement in proteostasis could potentially delay cognitive impairment. One emerging entry point to modulate proteostasis is the regulation of nucleo-cytoplasmic partitioning of proteins across the nuclear pore via karyopherins. The nuclear exportin XPO1 is a key regulator of proteostasis by driving the assembly of ribosomes and by modulating the process of autophagy. We recently found that the XPO1 inhibitor KPT-330 (Selinexor), an FDA-approved drug against multiple myelomas, enhances proteostasis, leading to benefits in models of neurodegenerative diseases in *C. elegans* and *Drosophila*. Here, we find that KPT-330 increases autophagy in murine neuronal cells. In a murine model of Alzheimer’s disease (5XFAD), KPT-330 improved spatial memory performance. Unexpectedly, general amyloid deposition in several brain regions was significantly increased by KPT-330, but specific regions, especially the thalamus, displayed significantly lower deposition, suggesting that XPO1 inhibition has regional-specific effects on proteostasis and amyloid plaque formation. Altogether, we conclude that, despite overall increases in amyloid plaque burden, XPO1 inhibition can improve cognition via spatially-specific reductions in amyloid deposition.

**Supplementary Information:**

The online version contains supplementary material available at https://doi.org/10.1007/s11357-026-02122-0.

## Introduction

Alzheimer’s disease (AD) remains a formidable challenge for biomedical researchers, with successful therapeutics currently restricted to antibodies targeting amyloid proteins [1], with debatable effectiveness and economic benefits [2]. At its core, AD is a progressive proteinopathy that burdens neuronal function and hinders survival to the point that cognition and memory are inevitably lost. As neurons are largely post-mitotic, cells manage proteome stability via proteostatic mechanisms that primarily rely on properly controlling protein synthesis, folding, and degradation machineries in order to maintain protein solubility and function. Post-mitotic systems such as the nematode *C. elegans* provide a useful model to study the biology of aging and the regulation of proteostasis. The vast majority of genetic models of enhanced proteostasis and longevity require active autophagy [3] and alterations in ribosomal assembly via nucleolar attrition [4]. Therefore, finding strategies that modulate these two key nodes of proteostasis may address the progressive proteostatic decline found in AD.

The nuclear export protein XPO1 (Exportin1, CRM1) is estimated to actively transport over 200 different proteins [5] containing nuclear export sequences (NES) across the nuclear pore, including tumor-suppressing proteins [6–8]. As such, slow reversible XPO1 inhibitors were developed to target the cysteine 528 in the NES-binding site of XPO1 [9, 10] and reduce the export of tumor-suppressing proteins, consequently preventing the loss of tumor-suppressing protein activity and reducing cancer cell proliferation [11]. Recently, the XPO1 inhibitor KPT-330 (Selinexor, XPOVIO) was approved against relapsed multiple myelomas [12]. XPO1 modulates the activity of the autophagy–lysosome pathway by controlling the localization of TFEB [8, 13]. Transcriptional activation of TFEB via XPO1 inhibition is accompanied by increases in lifespan in wild-type *C. elegans* and *Drosophila* afflicted with an ALS-related mutation. Loss of XPO1 activity also reduces overall rRNA levels, decreases ribosomal assembly, downregulates transcriptionally and translationally Fibrillarin, and restricts nucleolar size via autophagy protein GABARAP [14]. XPO1 inhibition therefore represents an emerging strategy to maintain proteome stability and function by leveraging different regulatory nodes of proteostasis.

In this study, we tested KPT-330 in cultured murine neuroblasts as well as a murine model of Alzheimer’s disease in order to evaluate its effect on the proteostatic decline accompanied by the progressive amyloid deposition, and its potential in improving cognitive functions. We find that KPT-330 prevents the loss of cognition in 5xFAD mice via region-specific reductions in amyloid deposition, suggesting that nuclear export inhibition can preserve key functionalities during the development of Alzheimer’s disease.

## Results

### KPT-330 enhances autophagy in murine neuroblastoma Neuro2a cells

**Fig. 1:**
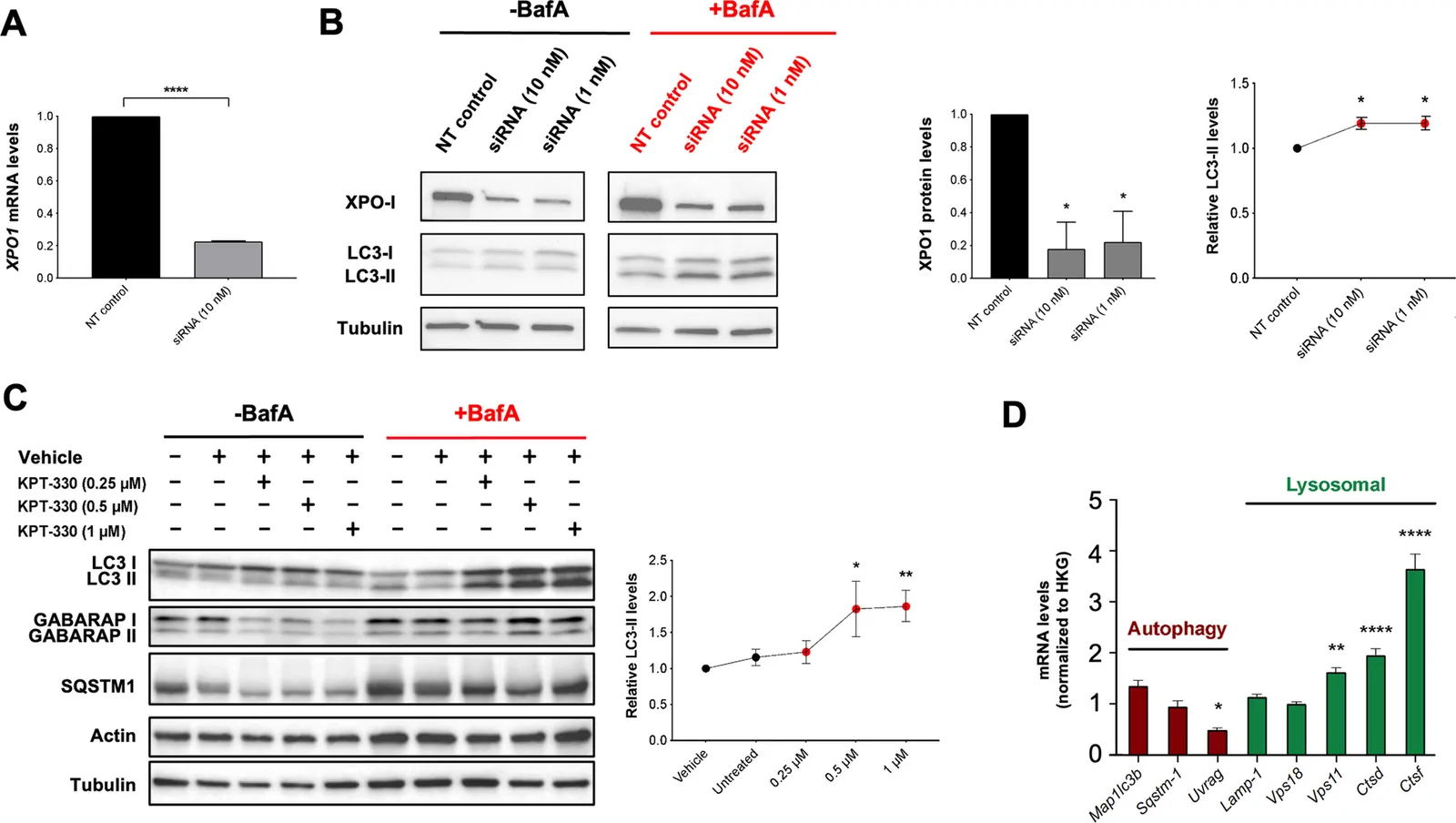
KPT-330 treatment increases autophagic degradation in Neuro2a cells. **A** *XPO*1 silencing efficiency was measured by qPCR (*n* = 3). **B** Proteins in control and silenced Neuro2a cells were immunoblotted and levels of XPO1 and LC3 forms (with Bafilomycin A1) were quantified (*n* = 3, ± SD ANOVA, *P < 0.05 vs control). **C** Neuro2a cells incubated with different concentrations of KPT-330 for 48 h followed by a treatment with BafilomycinA1 for 6 h and proteins were resolved by immunoblotting. LC3 forms were quantified and normalized with Actin (*n* = 3, ± SD one-way ANOVA, *P < 0*.*05, **P < 0*.*01). **D** Expressions of autophagy and lysosomal genes (normalized to housekeeping genes, HKG) in cells treated with vehicle (ethanol) or KPT-330 (1 µM) were quantified by qPCR (*n* = 3, ± *SD* *P < 0*.*05, **P < 0*.*01, ****P < 0*.*0001,* t*-test)

Reducing the activity or the expression of XPO1 was previously demonstrated to enhance proteostasis, autophagy, and lifespan in model organisms [8, 13]. Therefore, we sought to evaluate the impact of the loss of XPO1 activity on the onset of Alzheimer’s disease, a neurodegenerative disease characterized by autophagic dysfunction and proteostatic collapse. To reduce XPO1 activity, we opted to use KPT-330 (Selinexor), an FDA-approved XPO1 inhibitor [15] of relatively small size (443 Da) that can cross the blood–brain barrier. To assess the impact of XPO1 inhibition on autophagic degradation, we initially tested the effect of silencing *XPO*1 in murine neuroblastoma Neuro2a cells. *XPO*1 silencing led to significant reductions in both its transcript and protein levels (Fig. 1A–B). Using Bafilomycin A1 to disrupt lysosomal function, we found that the loss of XPO1 activity led to increases in LC3 turnover, suggesting higher rates of autophagic flux (Fig. 1B–C). Neuro2a cells treated with KPT-330 displayed enhanced lysosomal degradation of autophagy proteins LC3, SQSTM1, and GABARAP (Fig. 1C) [14]. Transcriptionally, some lysosomal gene targets of the major autophagy transcription factor TFEB were induced, suggesting improvements in lysosomal activity (Fig. 1D), as previously shown in HeLa cells [8]. Altogether, our data suggested that KPT-330 could potentially improve proteostasis in mice neurons by stimulating the autophagy–lysosome pathway.

### Chronic KPT-330 treatment improves cognition in 5XFAD Alzheimer mice

**Fig. 2:**
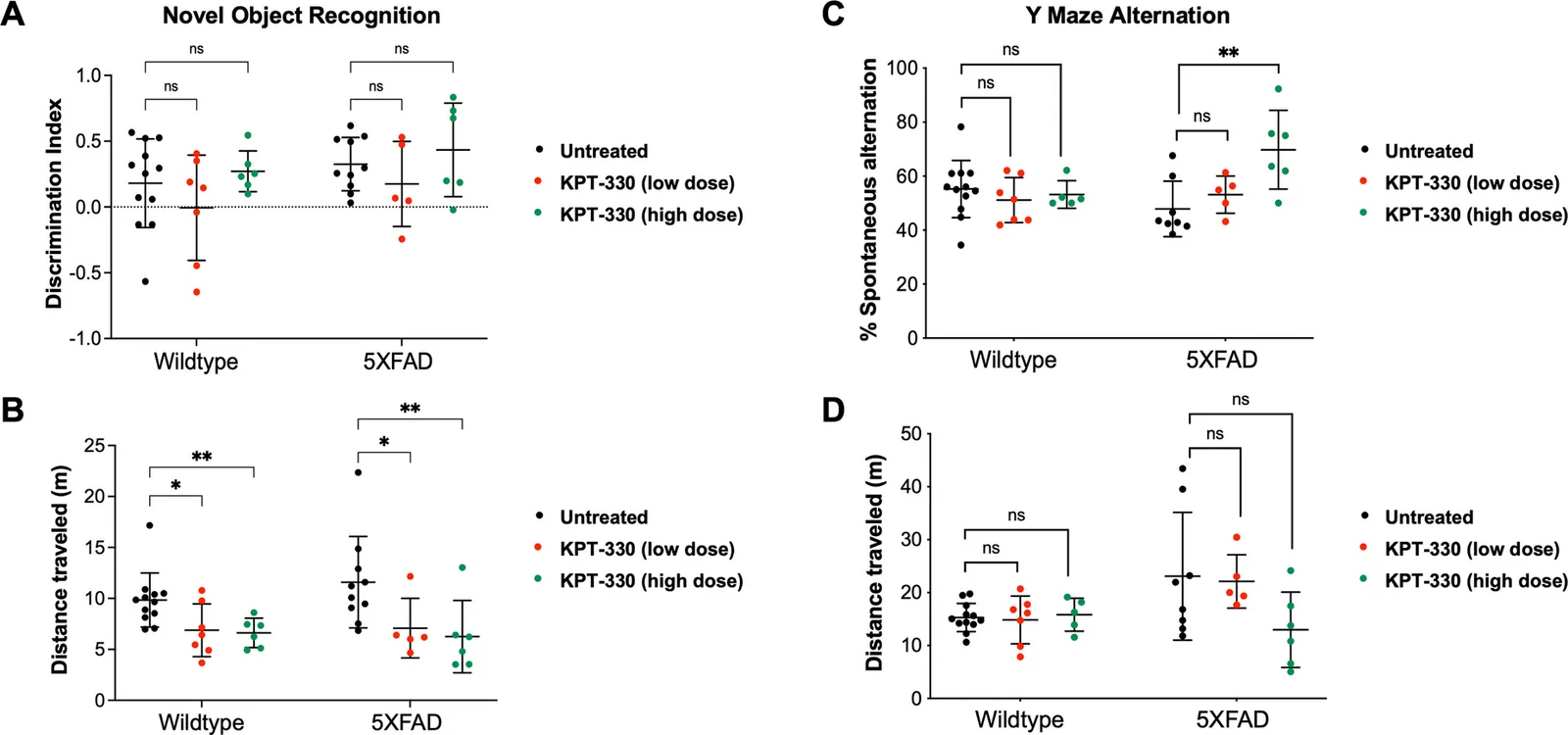
KPT-330 treatment improves cognition in 5XFAD mice performing the spontaneous alternation Y-maze test. **A** Wild-type and 5XFAD mice fed control chow or chow containing low or high doses of KPT-330 were subjected to novel object recognition where discrimination; **B** distance was measured at 6 months of age. **C** Spontaneous alternation and **D** distance traveled in a Y-maze were measured in 12-month-old wild-type and 5XFAD mice treated with or without KPT-330 for 8 months. Spontaneous alternation between untreated WT and 5XFAD mice was decreased by 13% (*P*, 0.1407) in other comparisons between untreated WT and 5XFAD for not significant; ns, not significant; *P < 0*.*05, **P < 0*.*01,* n* = 6*–*12, ± SD, one-way ANOVA

As KPT-330 is expected to cross the blood–brain barrier, we predicted that feeding KPT-330, an orally bioavailable compound [16], would lead to bioaccumulation in the brain. Using mass spectrometry, we found that KPT-330 readily accumulated in the blood and liver as well as the brain of C57BL/6 J mice (Figure S1A**-**C). To test the effects of XPO1 inhibition on Alzheimer’s disease onset, we fed KPT-330 (low dose 2.6 mg/kg/week or high dose 45 mg/kg/week) (Figure S1C) [17] in C57BL/6 J mice and the Alzheimer’s disease 5XFAD mice (hemizygous) [18] from 4 to 12 months of age. The rationale for the lower dose was to evaluate the impact of modest inhibition of XPO1, which could mimic conditions observed in long-lived nematodes that display partial reductions in XPO1 expression [13]. Chronic feeding of KPT-330 had minimal effect on weight (Figure S1D) and no discernible tolerability issue. We tested cognition when amyloid deposition is established [19] via two complementary cognitive evaluations, the novel object recognition (NOR) for recognition memory [20] and spontaneous alternation in Y-maze for spatial learning and memory. NOR testing revealed that KPT-330 did not improve novel object recognition (Fig. 2A–B and Figure S2A-C) [19]. Notably, the NOR test results remained relatively similar between WT and 5XFAD mice at 6 months of age. To capture clearer cognitive decline, we performed the Y-maze test at 12 months of age, initially finding at 9 months of age that spatial memory was decreased (− 27% spontaneous alternation) in untreated 5XFAD mice (Figure S2D). While we observed a slightly lower difference in spatial cognition (− 13% spontaneous alternation) at 12 months of age between untreated WT and 5XFAD mice, Y-maze testing showed that a high dose of KPT-330 significantly increased spontaneous alternations in 5XFAD but not in WT mice, suggesting an improvement in spatial memory in these mice (Fig. 2C–D). Both NOR and Y-maze tests showed tendencies for reduced overall distance traveled in 5XFAD mice treated with the high dose of KPT-330 (Fig. 2B and D and Figure S2E), suggesting that these generally hyperactive animals [21] may be less active with KPT-330 treatment. Taken together, KPT-330 treatment resulted in functional improvements in spatial but not recognition memory, suggesting positive effects of XPO1 inhibition on spatial memory deficits in an Alzheimer’s disease model that may arise from improved brain proteostasis.

### KPT-330 treatment does not affect vascular amyloid deposition

**Fig. 3:**
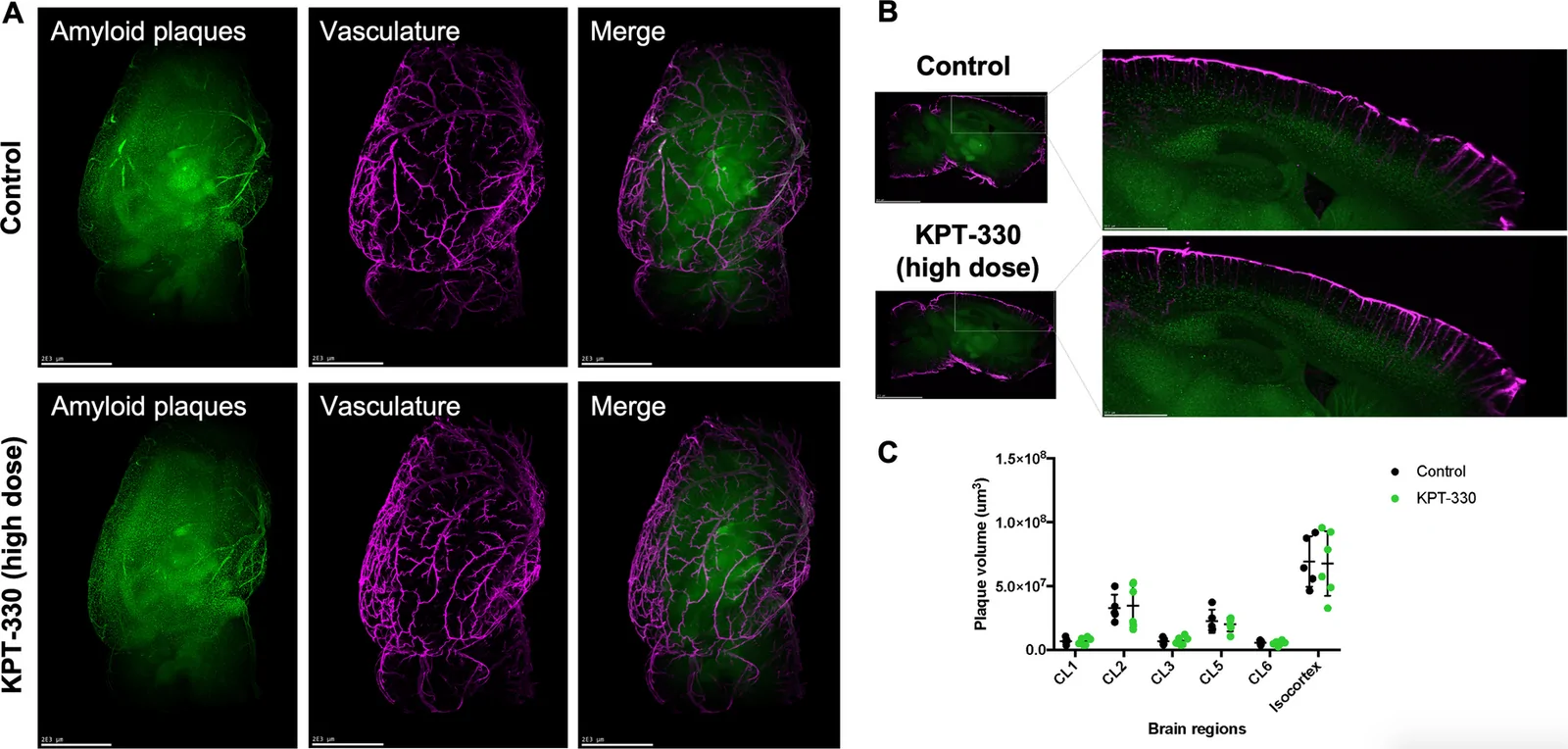
KPT-330 treatment does not affect vascular amyloid deposition. **A** Representative images of 5XFAD mice were treated with or without KPT-330 (high dose; 45 mg/kg/week) for 8 months (green, Congo red; purple, anti-transgelin). **B** Representative sagittal images of the hemisphere staining of vasculature and amyloid plaques; **C** quantification of amyloid plaque volume in vasculature of 6 brain regions: cortical layer (CL) 1, 2, 3, 5, and 6, and isocortex (*n* = 5–6 condition, ± SD, *t*-test). See supplemental videos S1–2 for 3D details

In order to fully characterize amyloid deposition in 5XFAD brains treated with KPT-330, we opted to visualize and quantify hemisphere-wide distribution of vascular and parenchymal amyloid deposition and plaques using 3D immuno-labeling and imaging via light sheet fluorescence microscopy. As amyloid buildup in vasculature may be indicative of impaired clearance [22], we compared amyloid deposition between control and high dose KPT-330 treated animals as we observed positive cognitive effects from high dose treatment. In both groups, plaque deposition was apparent throughout the cortex, in particular in deeper cortical layers, including isocortex layer 5. Overall, we observed minimal changes in vascular amyloid accumulation between control and treated mice (Fig. 3A–C, Data S1, and Videos S1-2) suggesting that the impact of KPT-330 in 5XFAD mice may specifically reside in brain regions relevant for cognition.

### 5XFAD mice treated with KPT-330 differentially accumulate amyloid depots

**Fig. 4:**
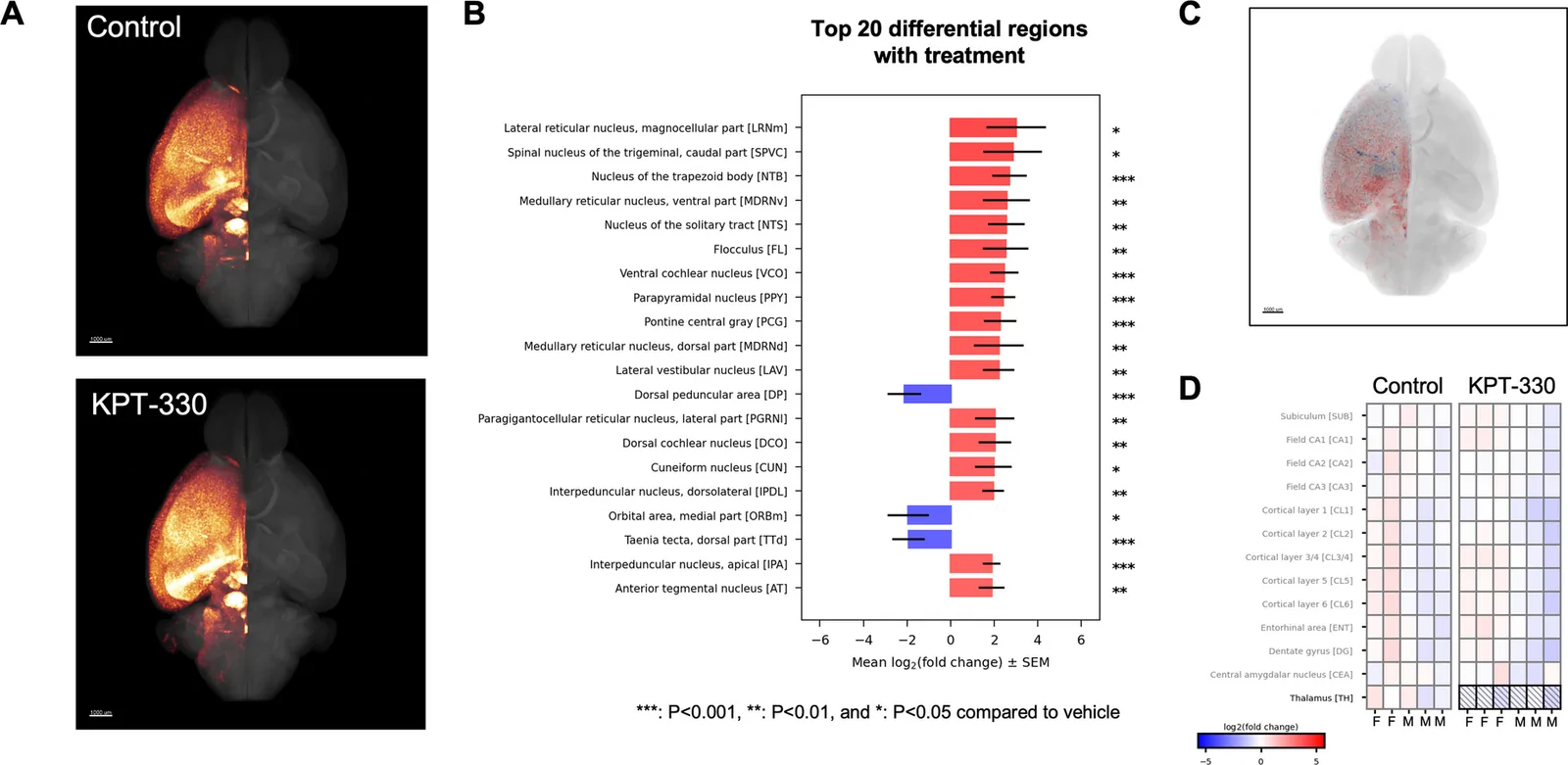
Amyloid differentially accumulates in the brain of 5XFAD mice treated with KPT-330. **A** Representative group average of amyloid Congo Red staining (*n* = 5–6/condition) in glow scale of brains from control and KPT-330 (high-dose; 45 mg/kg/week) treated 5XFAD mice. Scale bar = 1 mm. **B** Top 20 significantly differential brain regions for amyloid deposition (*n* = 5–6/condition, *t*-test, ± SD) ***P < 0.001, **P < 0.01, and *P < 0.05 compared to control, visualized in (**C**). **D** Amyloid deposition in 13 selected brain regions reveals significant reduction in the thalamus, highlighted: *P* < 0.05, *n* = 5–6 per condition, *t*-test

By imaging and quantifying amyloid deposition in various brain regions, we unexpectedly observed an overall increase in amyloid plaques in the brain of 5XFAD treated with KPT-330, with deposition found in several auditory-related regions (Fig. 4A–C and Figure S3, Data S1, Videos S1-2). Accumulated amyloid plaque volume was significantly increased in several brain regions following treatment with KPT-330, including the magnocellular part of the lateral reticular nucleus, the caudal part of the spinal nucleus trigeminal, the nucleus of the trapezoid body, the ventral part of the medullary reticular nucleus, and the nucleus of the solitary tract. These unexpected amyloid accumulations were accompanied by higher overall ubiquitinated protein levels, suggesting potential regional proteostatic burden (Figure S4A-B). However, some brain regions had significantly lower amyloid levels (Fig. 4B), including the dorsal peduncular area, a site recently linked to anxiety-like behavior [23]. Further analysis in 12 relevant areas for amyloid deposition showed that thalamic regions displayed significantly lower amyloid accumulation in 5XFAD animals treated with high-dose KPT-330 (Fig. 4D), suggesting that XPO1 inhibition causes differential amyloid deposition in the brain that protects decision-making regions. Finally, brain mRNA levels of autophagy genes LC3 and SQSTM1 were modestly increased by KPT-330 treatment (Figure S4B-C), in line with a transcriptional contribution to autophagy modulation by XPO1 (Fig. 1D, [8, 13]). However, KPT-330 did not globally impact the levels of neuroinflammatory proteins GFAP and Iba1 (Figure S4E). Taken together, our study shows that KPT-330 treatment impacts amyloid deposition in a region-specific manner and delays cognitive decline in a murine model of Alzheimer’s disease.

## Discussion

Therapeutic development against Alzheimer’s disease remains challenging, and approaches to enhance neuronal proteostasis have yet to be approved clinically. Here, we evaluated the effect of XPO1 inhibition in murine cells and a murine model of Alzheimer’s disease and uncovered that loss of XPO1 leads to improvement in some cognitive domains. While amyloid plaque build-up is correlated with neuronal loss and progressive cognitive decline, loss of XPO1 activity generates differential amyloid depositions across regions of the brain that ultimately favor cognition-relevant areas.

Modulating autophagy and lysosomal biogenesis are potential approaches to enhance the clearance of proteotoxic proteins found in Alzheimer’s disease [24, 25]. Inhibition of XPO1 with KPT-330 leads to improvements in the autophagic turnover rate and lysosomal biogenesis in cells and shows promising effects in improving proteostasis and survival in models of neurodegenerative disease in small organisms [8]. Here, we find that acute inhibition of XPO1 in Neuro2a murine neuroblastoma cells also enhances autophagic flux. We anticipated that this effect may be recapitulated in the brains of Alzheimer’s disease mice treated with KPT-330. Y-maze analyses showed that spatial cognition is improved with KPT-330 at a high dose, suggesting functional improvements across the brain and perhaps more specifically in regions important for cognition. However, our whole-brain analysis revealed limited increases in autophagy gene expression and protein levels with KPT-330 treatment. The changes in amyloid deposition varied widely across different regions in the brain, indicating region-specific responses to XPO1 inhibition, and unexpected increases of plaque accumulation globally, which suggests regional variations in amyloid solubility when XPO1 is inhibited. Notably, KPT-330 had limited effects on vasculature amyloid deposition, suggesting that XPO1 inhibition affects plaque deposition directly in functional brain regions instead of via circulatory clearance. Our region-specific data (Fig. 4D) were in line with previously described baseline sex-specific amyloid deposition in the 5XFAD mice model, whereas female mice brains generally tend to display higher levels of amyloid deposition across different brain regions [26]. Significant increases in plaque accumulation in auditory regions were observed, a region of interest recently recognized in 5XFAD mice for amyloid deposition [27]. Notably, thalamic regions accumulated significantly less amyloid plaques when mice were treated with KPT-330, highlighting protection in a region recognized for behavioral and cognitive processing [28, 29]. Some neurological pathologies such as schizophrenia and multiple sclerosis, as well as traumatic brain injury, have been shown to weaken the blood–brain barrier at the thalamus [30–32]. Whether this is the case in the 5XFAD model is unclear, but it could conceivably contribute to localized thalamic differences in KPT-330 bioaccumulation and effects. Seemingly, XPO1 inhibition in the brain of 5XFAD mice affects proteostasis differentially, indicating that modulation of nuclear protein export can lead to region-specific outcomes in amyloid accumulation. The impact on proteostasis may also involve other known effects of XPO1 inhibition beyond autophagy, such as reduction in nucleolar size and ribosomal assembly, which could elicit stress responses in certain cell types and brain regions. However, the exact underlying mechanism for these differential effects remains to be elucidated. Also, whether amyloid plaque volume always correlates with neuronal dysfunction is unclear. Complex, regionally specific responses to XPO1 inhibition may account for our observations, and the level of XPO1 inhibition across different regions may vary due to different drug penetration. As KPT-330 is FDA-approved for relapsed myelomas [15], it would be interesting to investigate its impact on neurological functions in treated patients. Overall, our study provides unexpected and promising results on the benefits of XPO1 inhibition on cognition but also reveals the regional impact of KPT-330 on brain amyloid deposition. These spatially specific effects of XPO1 inhibition in the brain warrant further studies as they may suggest cell type-specific responses in different brain regions from the loss of XPO1-mediated nuclear export, unraveling region-specific proteostatic flexibility that has ramifications in the development of Alzheimer’s disease.

## Limitations

AD model used: While the 5XFAD is an accepted model for amyloid deposition, it lacks a Tau pathology component, which would add more translatability to human AD. Behavioral testing: While the Y-maze test provides strong evidence for improved spatial memory, additional behavioral analyses would further strengthen these findings. The number of mice was restricted by the prohibitive cost associated with a longitudinal study and following ethical considerations. 3D imaging of amyloid deposition: Quantification of amyloid plaque volume generally reflects disease progression, but Congo Red–positive plaque volume could also correspond to drug-induced sequestration of more toxic amyloid species into dense-core plaques. Drug penetrance: While our initial pharmacological analyses showed whole brain accumulation of KPT-330, regional drug accumulation may differ. Brain protein level determination: Protein analyses with whole-brain homogenates inadequately resolve regionally specific protein and autophagic changes. Spatially relevant modulation of proteostatic mechanisms by KPT-330 remains unclear. Region-specific plaques may also elicit different glial responses, and those may be modulated by XPO1 inhibition. XPO1 inhibition: Varying levels of XPO1 activity across different brain regions from KPT-330 treatment may generate distinct proteostatic responses that could contribute to differences in amyloid deposition.

## Methods

### Immunoblotting in cells and tissues

Cells were washed with PBS, and protein lysates were collected using RIPA buffer (50 mM Tris–HCl, 250 mM sucrose, 1 mM EDTA, and Roche protease inhibitor tablet, with 5% SDS) and subjected to heat for 10 min. Tissues (10 mg) were homogenized with a pre-chilled Dounce homogenizer using RIPA buffer (50 mM Tris–HCl, 250 mM sucrose, 1 mM EDTA, and Roche protease inhibitor tablet). Tissue lysates were cleared by cold centrifugation. Lysate protein concentrations were quantified using the DC Protein Assay kit (BIO-RAD). Proteins were separated by SDS-PAGE on a 4–15% Tris–Glycine gel and transferred to nitrocellulose. The membrane was briefly stained with Ponceau S to confirm even transfer, rinsed with TBS-T until clear, blocked with 5% nonfat dry milk in TBS-T, and immunoblotted with anti-LC3 (Abcam, ab51520), anti-SQSTM1 (Abcam, ab211324), anti-GABARAP (Abcam, ab109364), anti-Ubiquitin (ThermoFisher MAI-10035), anti-Tubulin (Abcam, ab6160), and anti-Actin (Milllipore, MAB1501R), anti-GAPDH (Cell Signaling, 5174S), anti-GFAP (Proteintech, 16825-1-AP), and anti-Iba1(Abcam, ab5076). Proteins were revealed using SuperSignal West Femto Maximum Sensitivity Substrate (ThermoFisher) and imaged on a ChemiDoc Imaging System (BIO-RAD). Densitometric analyses were conducted with ImageJ (NIH), and statistical analyses were performed using the Prism 7 software (GraphPad).

### mRNA extraction and analysis by qRT-PCR

Tissue and cell mRNA was extracted using Trizol Reagent and purified thereafter using Qiagen RNeasy kit. To perform qRT-PCR, cDNA was prepared as previously described [33]. qRT-PCR was performed using the QuantStudio 5 Real-Time PCR System (Applied Biosystems, ThermoFisher) and PowerTrack SYBR Green Master Mix (Applied Biosystems, ThermoFisher). The thermal cycling conditions were as follows: an initial denaturation at 95 °C for 10 min, followed by 40 cycles of denaturation at 95 °C for 15 s and annealing–extension at 60 °C for 1 min. Melt curve analysis was performed at the end of the reaction to verify the specificity of amplification. Data were analyzed using QuantStudio 5 software (Applied Biosystems, ThermoFisher). Expression of housekeeping genes (HKG) *B*2*m*, *Actb*, and *Gapdh* was measured, and genes of interest were normalized by the geometric mean of HKG as previously described [33]. Primer sequences can be found in Supplemental Table S1. The Prism 7 software (GraphPad) was used to conduct statistical analyses.

### Pharmacological analysis of KPT-330

KPT-330 (Adooq Bioscience) and fentanyl D5 (Millipore Sigma) super stock solutions were prepared in methanol at a concentration of 1 mg/ml. A working stock solution of KPT-330 was prepared each day from the super stock solutions at a concentration of 10 µg/ml and used to spike the calibrators. The LC/MS/MS system consisted of a Shimadzu SIL 20 A HT autosampler, LC-20AD pumps, and an AB Sciex API 3200 tandem mass spectrometer with turbo ion spray. The LC analytical column was an ACE Excel C18-AR (75 × 3.0 mm, 3 micron, Mac-Mod Analytical) and was maintained at 25 °C during the chromatographic runs using a Shimadzu CT-20A column oven. Mobile phase A contained 0.1% formic acid dissolved in water. Mobile phase B contained 0.1% formic acid dissolved in 100% HPLC grade acetonitrile. The flow rate of the mobile phase was 0.4 ml/min. KPT-330 and fentanyl D5 (the internal standard) were eluted with a gradient. The initial mobile phase was 25% B and, at 1 min after injection, was ramped to 100% B. From 5.0 to 8.0 min, the mobile phase was maintained at 100% B, and at 8.01 min, it was switched immediately back to 25% B and ran for 1.99 min to equilibrate the column before the next injection. The KPT-330 transition was detected in positive mode at 444/334.1 Da. The fentanyl D5 transition was detected at 342/188 Da.

In order to determine bioaccumulation of KPT-330, 4-month-old mice received a bolus dose of KPT-330 (p.o.) and were then sacrificed at 60, 180, 360, 540, and 720 min post administration. Blood, brain, and liver were collected and assessed for KPT-330 concentration in each mouse. At each time point, *n* = 3 male and *n* = 3 female mice were included. KPT-330 was suspended in a solution of 5%Ethanol/0.6% Gibco® Pluronic® F-68/0.6% Polyvinylpyrrolidone (K30). KPT-330 was quantified in mouse whole blood. Calibrator samples were prepared daily by spiking blank blood to achieve final concentrations of 0, 1, 5, 10, 50, 100, and 500 ng/mL. Briefly, 0.1 mL of calibrator and unknown blood samples were mixed with 10 µL of 1 µg/mL fentanyl D5 and 1 mL of methanol. The samples were vortexed vigorously and then centrifuged at 13,000 g for 5 min at 25 °C. The supernatants were transferred to a new 3 mL tube and dried to residue under a gentle nitrogen stream. The residue was then redissolved in 100 µL of 50/50 mobile phase A/mobile phase B, vortexing for 30 s. The samples were transferred to injection vials and 10 µL was injected into the LC/MS/MS. The ratios of KPT-330 peak areas to fentanyl D5 peak areas for each unknown sample were compared against a linear regression of the ratios obtained by the calibration samples to quantify KPT-330. The concentration of KPT-330 was expressed as ng/mL blood. Each brain and liver were weighed in a polypropylene tube, and then a 10 × volume of 75% methanol was added. Each sample was thoroughly homogenized. Calibrator samples were prepared daily by spiking a mixture of blank male and female control homogenate of each organ type to achieve final concentrations of 0, 1, 5, 10, 50, 100, and 500 ng/mL. The samples were prepared for injection and KPT-330 was detected as described above. The concentration of KPT-330 was expressed as pg/mg of tissue.

### Mice maintenance, breeding, and behavioral testing

Mice were raised on regular chow (NIH-31, Envigo), and 5XFAD hemizygous males were bred with B6J females (2 females per male ratio), and pups were raised and weaned by 21 days of age. Mice were fed control, low dose, or high dose KPT-330 from 4 months of age onward. KPT-330 (Selleckchem) was added directly to the food mixture and food pellet (Envigo). Wildtype and 5XFAD mice on control or KPT-330 chow were assessed for cognitive performance via the novel object recognition (NOR) and spontaneous alternation Y-maze tests at 6 and 12 months of age, respectively, using the ANY-Maze video tracking and analysis software. Prior to testing, all animals were habituated to operator handling for 3 consecutive days at 1 min per animal during h of the animals’ light cycle. On testing days, animals of different sexes were separately habituated in test rooms for 30 min prior to the start of testing. All tests were conducted under low noise and lighting conditions (less than 30 lx) during hours of the light cycle. All testing arenas and objects used were cleaned thoroughly with chlorhexidine and air dried to remove scent between animals. Animals which were tested were transferred to holding cages to reduce behavioral effects on untested littermates, which may confound test readouts.

NOR assessment was adapted from [20]. Briefly, animals were first individually subjected to 30 min of habituation to an empty test arena approximately 30 × 30 × 30 cm, during which an open field test was concurrently performed to assess activity and anxiety levels (day 1). Animals were removed from the test arena and subjected to object familiarization 24 h later (day 2). In this familiarization phase, animals were exposed to 2 identical objects placed at different corners of the test arena for 10 min, during which object bias was determined by the following metric:

$${\mathrm{Object\;bias\;score\:}}=\:(time\;at\;object1/time\;at\;both\;objects){\mathrm{\:\times\:100\%}}$$

Animals exhibiting an object bias score below 20% or above 80% were excluded from subsequent analysis. In the test phase (day 3; 24 h later), animals were returned to the test arena with one familiar object from the previous day and one novel object, both at the same positions as before, for 10 min. The ability of the animals to distinguish novel from familiar objects was determined by two complementary measures, novelty preference (%) and discrimination index:

$${\mathrm{Novelty\; preference}} = (Time\;at\;novel\;object/Time\;at\;both\;objects) {\mathrm {\:\times \;100\%}}$$

$${\mathrm{Object\; Bias\;Score}}=(Time\;at\;novel\;object-Time\;at\;familiar\;object)/{\mathrm{Time\;at\;both\;objects}}$$

Preferential interaction with novel objects is suggestive of recognition for previously encountered familiar objects and is indicated with preference scores above 50%, whereas scores of 0–50% indicate no novel object preference. Similarly, positive and negative discrimination indexes indicate novel object and familiar object preferences, respectively. Object interaction is scored if the animal was within 2 cm of it in the absence of object mounting.

The spontaneous alternation Y-maze test was previously described in [34]. Briefly, animals were placed into the middle of the starting arm and allowed to explore all arms of the maze for 8 min. Entry into an arm is scored when all four paws of the animals are inside the arm. For a spontaneous alternation to occur, the animal must enter a different arm of the maze consecutively and is calculated as follows:

$${\mathrm{Spontaneous\;alternation}} = (Number\;of\;spontaneous\; alternations/Total\; arm\; entries - 2)\;{\mathrm{\times\; 100\%}}$$

Increasing percentage of spontaneous alternations is suggestive of enhanced spatial memory. Notably, behavioral data normality was evaluated and confirmed by the Shapiro–Wilk test using the Prism 7 software (GraphPad).

### Brain imaging and analysis

Brains were dissected and fixed by perfusion with Neutral Buffered Formalin. Intact hemispheres were stained using an adapted version of the previously published protocol for immunolabelling-enabled 3D imaging of solvent cleared organs (iDISCO) [35]. Beta-amyloid plaques were stained using Congo Red (15 µM, #C6277, BioXtra, Sigma Aldrich), and the vasculature was immunostained with an anti-transgelin-targeting (SM22) antibody (1:1000, ab14106, Abcam) and donkey anti-rabbit (1:1000, 711–655-152, Jackson ImmunoResearch). Following tissue clearing, the intact hemispheres were imaged using a light sheet fluorescence microscope (LSFM) (UltraMicropscope II, Miltenyi Biotec GmbH, Bergisch Gladbach, Germany) (Gubra). The hemispheres were positioned in a sagittal orientation within a dibenzyl ether-filled chamber for refractive index matching. Double-sided illumination was applied with an exposure time of 232 ms, capturing images at 10 µm intervals at × 1.2 total magnification. Emission wavelengths of 500 nm (tissue autofluorescence), 560 nm (Congo Red), and 785 nm (SM22) were used for data acquisition. For each sample, hemisphere-wide plaque distribution was detected by Congo Red, and the plaque volume was mapped to one of more than 800 + unique brain regions in a LSFM optimized reference atlas [36]. Plaques were divided into two populations depending on the distance to a blood vessel; > 3 µm distance categorizes as parenchymal, and < 3 µm categorizes as vascular. Regions expressing significant regulation were presented via bar plots of plaque volume and group-wise statistics. All region names and abbreviations follow Allen’s Brain Atlas nomenclature. For each region, a negative binomial generalized linear model (GLM) was fitted to the quantitative data, and a subsequent Dunnett’s test was performed for multiple comparisons. Significantly regulated regions went through statistical validation, where the model deviance residuals were investigated to see if they aligned with the assumptions of Normality and homoscedasticity. Cook’s distance was evaluated for each data point to make sure no point is overly influential in the fitted model. If a region did not pass the validation, it was not considered significant. Notably, significance may be affected by the integrity of brain regions (i.e., dissection) and by the abundance of plaque in the region (no lower threshold was set on plaque size and volume, meaning that few plaques in small regions can drive significance). Plaque volume was quantified in individual brain regions; further, the vascular plaque volume was determined by layer-wise analysis in the isocortex. The effect of treatment between groups was assessed in 12 preselected regions by statistical analysis.

## Supplementary Information

Below is the link to the electronic supplementary material.

ESM 1 MP4 (40 MB)

ESM 2 MP4 (40 MB)

ESM 3 PDF (3.54 MB)

ESM 4 XLSX (79.9 KB)

## Data Availability

Requests about data and resources from this study can be sent to Louis R. Lapierre (louis.rene.lapierre@umoncton.ca).
