# Supplementary material for: Spatial memory in Alzheimer’s disease 5XFAD mice is enhanced by XPO1 inhibitor KPT-330
Source: GeroScience. 2026 Feb 6;48(6):8341–52. doi: 10.1007/s11357-026-02122-0 (PMC13638857; doi:10.1007/s11357-026-02122-0)
Supplement: Supplementary file 1 [file 11357_2026_2122_MOESM3_ESM.pdf]

Figure S1.

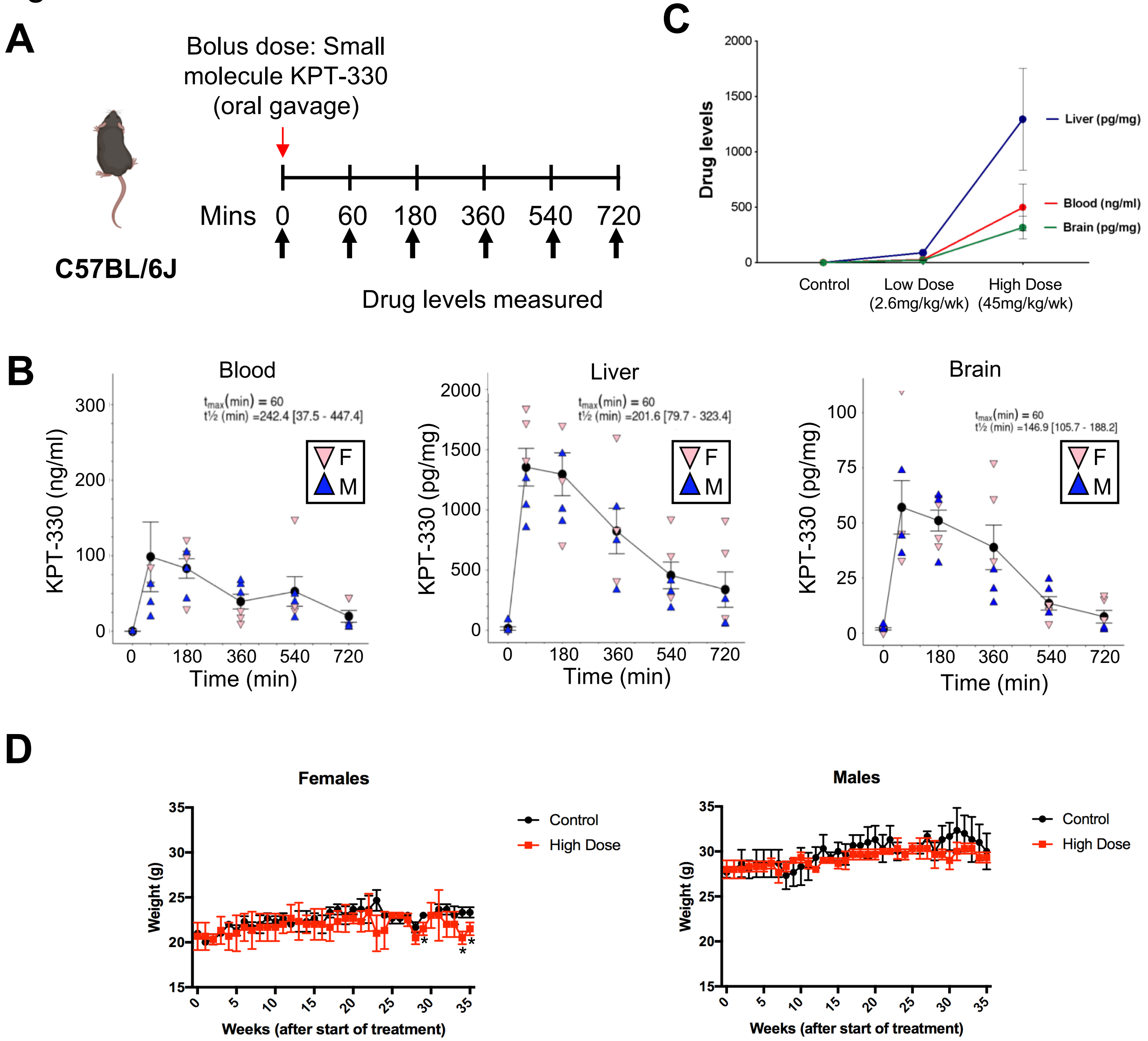

Figure S1. Pharmacological and bioaccumulation analysis of KPT-330. A. C57/B6 mice received a bolus dose of 7.5mg/kg KPT-330 (p.o.) and were then sacrificed at 60, 180, 360, 540, and 720 min post administration. **B.** Blood, liver and brain were collected and assessed for KPT-330 concentration in each mouse. At each time point, 4-month old male (n=3) and female (n=3) mice were included. **C.** Blood, liver, and brain levels of KPT-330 were also analyzed in 4-month old mice fed chow, or chow with low or high dose of KPT-330 for two weeks. **D.** 5XFAD mice weight was measured for the duration of the treatment (45mg/kg/wk) from 4-months to 12 months of age (n=3 per sex per treatment) ±*SD *: P<0.05, t-test*.

Figure S2.

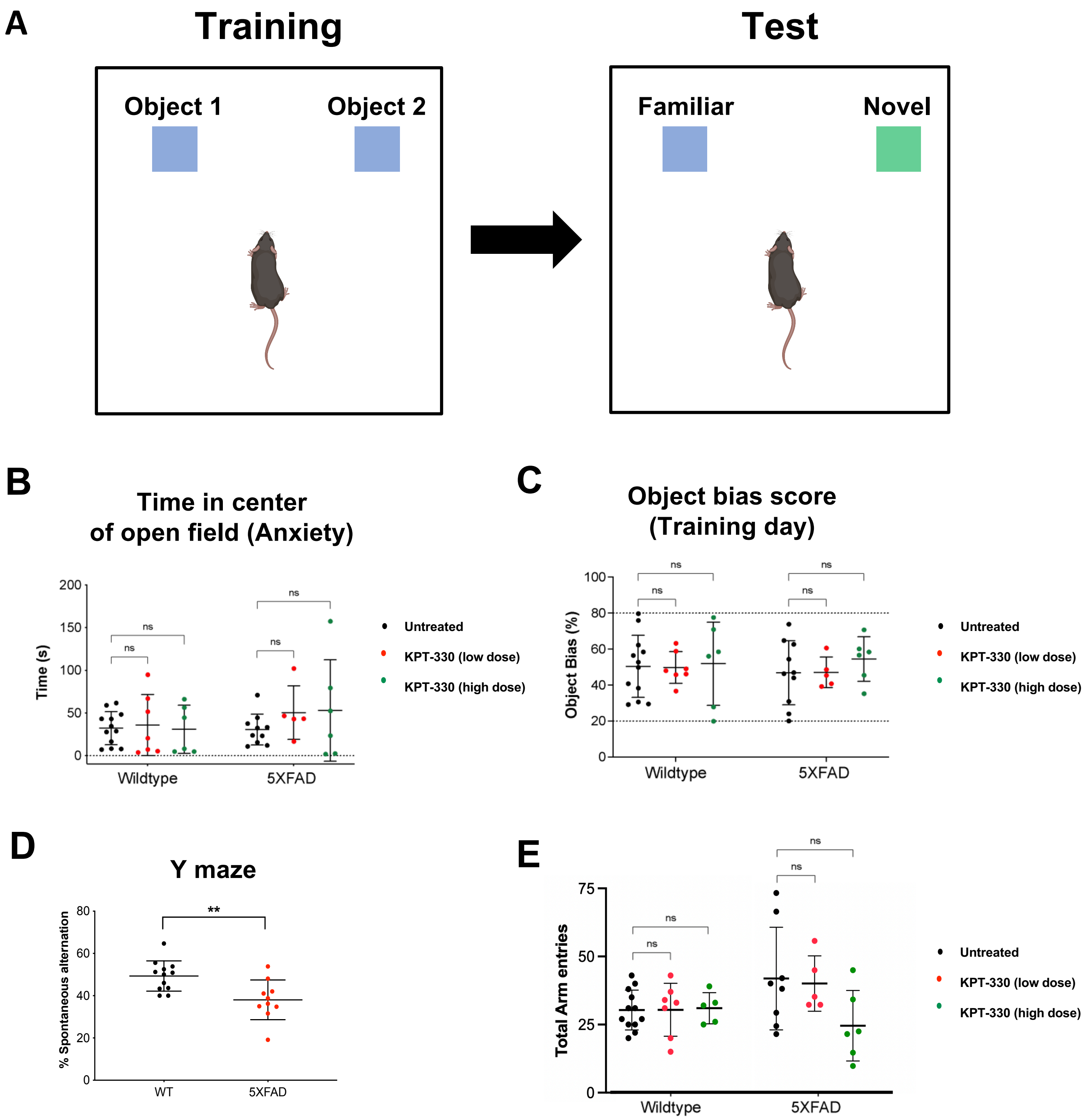

Figure S2. Novel object recognition (NOR) and Y-maze testing in 5XFAD mice. A. Four-month old wildtype and 5XFAD mice were fed with either chow or chow containing high (45 mg/kg/wk) or low (2.6 mg/kg/wk) dose of the XPO1 inhibitor, KPT-330, for 2 months and assessed for effects on recognition memory using the NOR test at 6 months of age following prior habituation to the test arena and object familiarization (see methods for details). **B.** Time in center of open field and **C.** object bias score during training day were measured. **: *P<0.01*, ±*SD* n=8-12, *ANOVA.* **D.** 9 month-old wild type and 5XFAD were assessed for effects on spatial memory using the Y-maze test. ±*SD* n=6-12, *t-test.* **E.** Number of arm entries associated with the Y-maze test presented in Figure 2C-D. Other than in **D**, comparisons between untreated WT and 5XFAD were not significant. ns: not significant, ±*SD* n=5-12, *ANOVA.*

Figure S3.

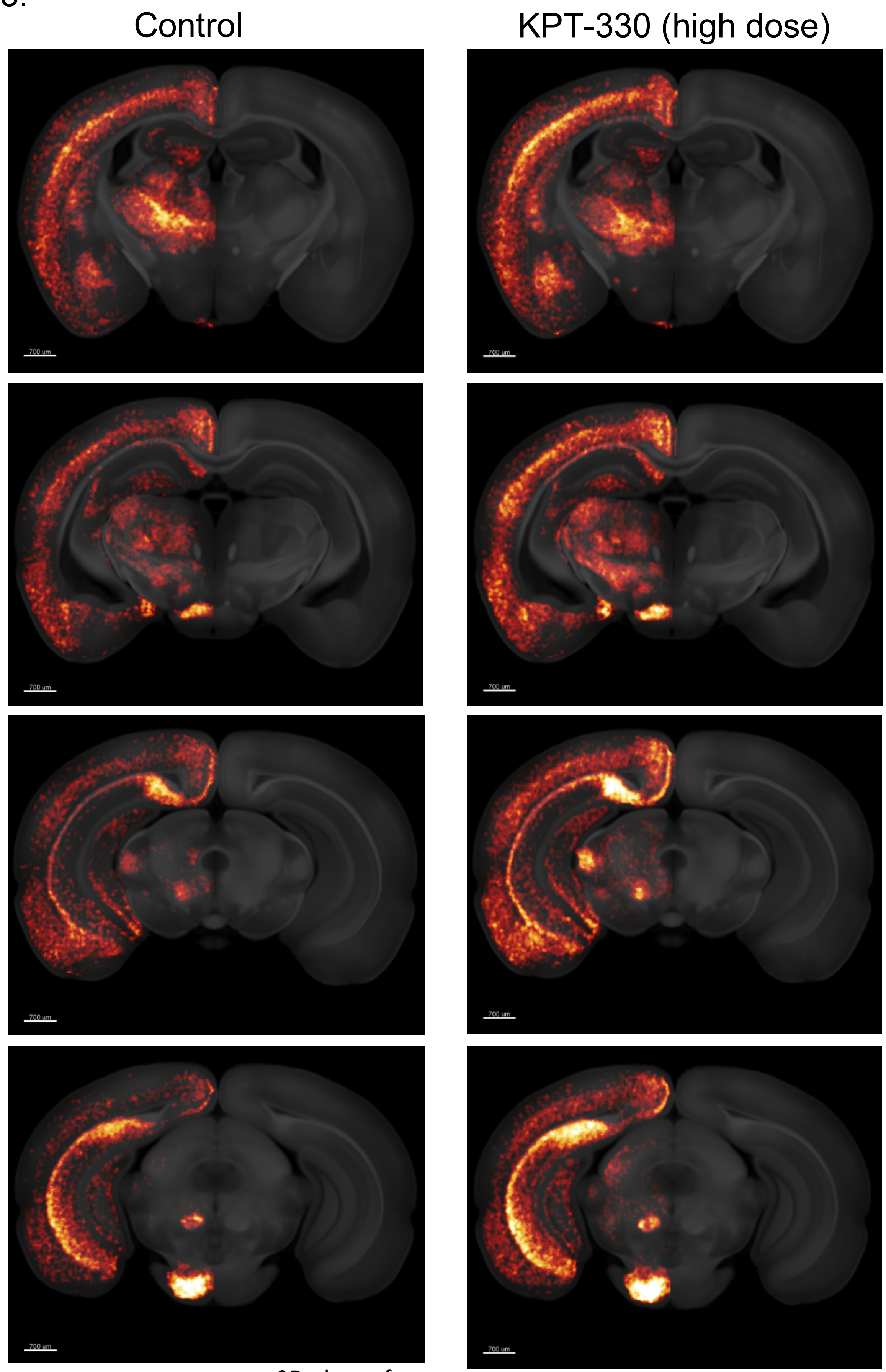

2D planes from average maps

Figure S3. Differential amyloid accumulation in 5XFAD mice brains. Representative 2D images at selected coronal planes showing group average of plaque volume in response to KPT-330 treatment and with control shown in glow scale. Scale bar 700 μm.

Figure S4.

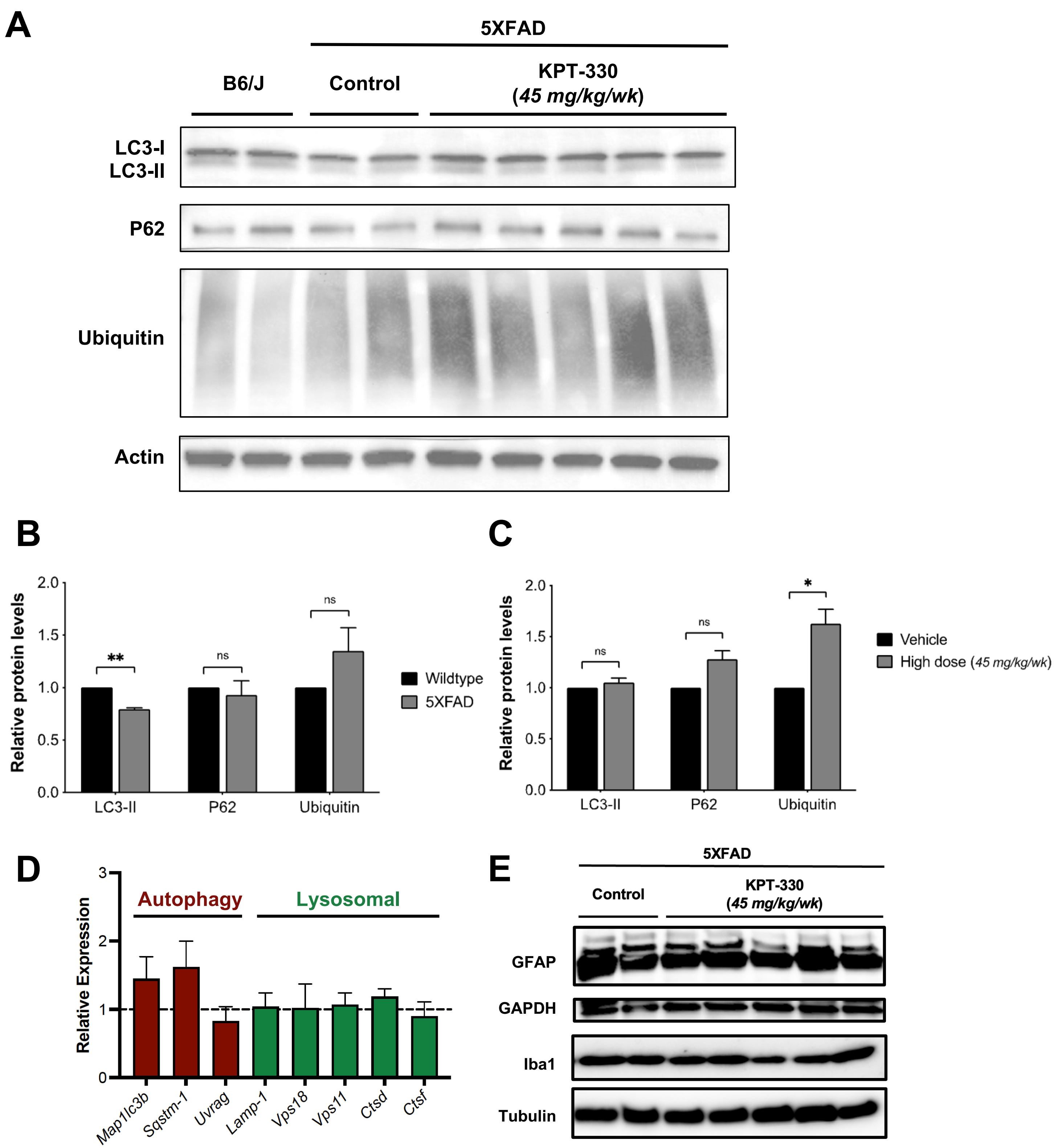

Figure S4. Protein analysis in brains of 5XFAD mice. A. Immunoblotting of LC3, p62 (SQSTM1) and Ubiquitin in brains of a group of 9-months old B6/J and transgenic 5XFAD mice (with or without KPT-330 from 4 months of age). **B.** Relative protein levels was compared between B6/J (wild-type) and 5XFAD (**B**) and between control (vehicle) and high dose KPT-330 in 5XFAD mice (**C**). Values expressed as mean of *n* = 2 ± SEM; *: *P<0.05,* **: *P<0.01.* **D.** mRNA levels of autophagy and lysosomal genes in the brain of 9-month-old 5XFAD animals and treated with KPT-330 compared to control (normalized at 1), ±*SD* n=3, *t-test*. **E.** Immunoblot of GFP, GAPDH, Iba1 and Tubulin in brains of a group of 9-months old B6/J and transgenic 5XFAD mice (with or without KPT-330 from 4 months of age), no significant differences, not shown.

Table S1.

Gene name	Sequence 5' -3'
Gapdh Forward	CATCACTGCCACCCAGAAGACTG
Gapdh Reverse	ATGCCAGTGAGCTTCCCGTTCAG
Actb Forward	GTGACGTTGACATCCGTAAAGA
Actb Reverse	GCCGGACTCATCGTACTCC
B2m Forward	ACCCGCCTCACATTGAAATCC
B2m Reverse	GGCGTATGTATCAGTCTCAGTG
Map1lc3b Forward	CGGACTGAGACACACACAAGG
Map1lc3b Reverse	TCGCTCTATAATCACTGGGATCT
Sqstm1 Forward	GGCCTATCTTCTGGGCAAGG
Sqstm1 Reverse	CCGGCACTCCTTCTTCTCTTT
Uvrag Forward	ATCCGACGTGGAGGAGTCTT
Uvrag Reverse	TGCGTTTGGATGACCCTTGT
Lamp 1 Forward	TGCTCCGGGATGCCACTAT
Lamp 1 Reverse	CTTGTTGTCCTTTTTCAGGTAGGTG
Vps18 Forward	CGCCTGCGTCCATGTCTATAA
Vps18 Reverse	GGCAGCACATCCTCGATCTT
Vps11 Forward	CTGGAAAAGAGAGACGGTGGCAATC
Vps11 Reverse	GAAAGGCCAGCCCAGTAACG
Ctsd Forward	CTGTGGGTCCCCTCCATTCATTG
Ctsd Reverse	GGTAGCCCATGCCCAAGATG
Ctsf Forward	CACAGCTCAGTATGGGATCACC
Ctsf Reverse	TTGGCTGGACTCATCTTCCTGC

Table S1. Primer used in the study

Data S1.

See Excel File

Video S1.

See Video File

Video S2.

See Video File
